# *Pleurotus* biomass production on vinasse and its potential use for aquaculture feed

**DOI:** 10.1080/21501203.2014.988769

**Published:** 2014-12-18

**Authors:** S.B. Sartori, L.F.R. Ferreira, T.G. Messias, G. Souza, G.B. Pompeu, R.T.R. Monteiro

**Affiliations:** a Escola Superior de Agricultura ‘Luiz de Queiroz’/USP, Av. Pádua Dias, 11, Piracicaba, SP, Brazil; b Centro de Energia Nuclear na Agricultura/USP, Caixa Postal 96. Av. Centenário, 303, Piracicaba, SP, Brazil

**Keywords:** basidiomycete, biomass, ration, *Danio rerio*, toxicology

## Abstract

The vinasse is a by-product generated during the manufacture of alcohol from sugarcane fermentation. Rich in organic matter, it is known that the vinasse has the potential to be used as a source of nutrients for plants as well as microorganisms. In this study, the fungi *Pleurotus sajor-caju, P. ostreatus, P. albidus* and *P. flabellatus* were cultivated in vinasse and utilised as a complementary diet for *Danio rerio* fish. The fungi mycelia cultured in vinasse for 15 days were lyophilised and offered to the fishes at a rate of 2% (medium/body weight) for 28 days. *P. albidus* produced the highest biomass (16.27 g L^−1^). Bromatological analysis of mycelia showed similar values to commercial rations. Toxicity tests showed that fish survival was 100% and no significant biomass loss was observed, indicating that the tested fungi grown in vinasse showed no toxicity. Our results showed that vinasse is a promising by-product for fungal growth and the mycelia of *Pleurotus* sp. fungi can be included in the diets of fish as a nutritional supplement.

## 1. Introduction

The anthropogenic intervention in the environment has revealed the performance of microorganisms in the pursuit of adaptation, when faced with damaging agents, thus becoming a powerful tool for environmental protection that can be further enhanced (Ferreira et al. 2010). Industrial effluents represent a source of environmental pollution of rivers, lakes and seas, and it can affect the water quality and the microbial and aquatic flora (Kanu & Achi 2011). Promising alternatives for solving numerous environmental problems caused by industrial activity can be derived from the study of new technologies for the treatment of industrial effluents.

Vinasse is a by-product derived from the production of alcohol, after pulp fermentation and distillation of wine. Between 8 and 15 L of vinasse is produced for each litre of alcohol manufactured (Ferreira et al. 2010). It has a dark-brownish colour and is also rich in nutrients. The effluent is characterised by a very high organic content, dark-brown colour, acidic nature (low pH) and a high concentration of untreated solids (Ferreira et al. 2011). Single cell-protein was produced from yeast growth using vinasse by Selim et al. (1991), and the addition of vinasse to the growth media of algae also presents a positive effect on the growth of cells, as observed for *Spirulina* (Barrocal et al. 2010).

Due to their reproduction and growth, fungi can adapt to a wide variety of substrates, allowing the bioconversion of residual substrates into microbial biomass. Fungal processing could potentially improve the economics and lessen the environmental impact of the biofuel industry, as the protein-rich fungal biomass can be formulated to produce high-value aquaculture and animal feeds, providing a market value close to $1190 per dry ton (Nitayavardhana & Khanal 2010).

Species of the genus *Pleurotus* occupy the third rank in the commercial production of edible mushrooms in the world, being found naturally in lignocellulolytic materials (Obodai et al. 2003). They have also been widely reported as foods with activities in many medical therapies and other related activities, including antitumor and antiviral activities and induction of immune activation (Yang et al. 2002), as well as antioxidant, antimicrobial, antimitogenic and antiproliferative activities (Ngai & Ng 2004). They represent low-calorie foods with a high water content (85–90%) (Manzi et al. 1999), containing 40–45% crude protein, 38–45% carbohydrates, 6–8% fibre, 5–7% ash and 3–4% lipids, and predominantly linoleic acid (70–78%). They also contain vitamins B (B1, B2) and C complex, niacin, biotin, a large amount of potassium (2.97%), and other minerals such as phosphorus (P), magnesium (Mg), calcium (Ca), sodium (Na), copper (Cu), zinc (Zn), iron (Fe), manganese (Mn) and molybdenum (Mo) (Furlani & Godoy 2005).

Constituents such as vitamins, proteins, lipids and fibres are the focus of professionals in the areas of health and food, since they play important functions in human and animal physiology. In several species of *Pleurotus*, carbohydrates and proteins are major components, followed by minerals, combined with a low fat content in most species (Furlani & Godoy 2005).

Furthermore, fungi of the genus *Pleurotus* provide easy management and production and grow very fast and with small footprints, using cheap and easily available raw materials (straw, grass and bagasse) as support media; they also have resistance to pests and diseases (Rajarathnam et al. 1992). Taking into account these advantages and traits of their cultivation and nutritional characteristics, fungi of the genus *Pleurotus* can be used as a natural food or food supplement in the diets of many animals that are important for human consumption, since they offer fast, cheap production.

According to the National Association of Manufacturers of Food for Animals – Anfalpet (2000), studies of natural feeds aim to assist in developing strategies for the sustainable management of ecosystems, thus helping ecologists, fisheries managers and fish culturists. Among the various aspects related to pisciculture, those involved with food have been widely discussed, mainly because they represent about 70% of the production costs in intensive cropping systems.

*Danio rerio*, also known as zebrafish, is a tropical freshwater fish belonging to the family Cyprinidae, Order Cypriniformes (Froese & Pauly 2007). This fish is omnivorous, having as its main food zooplankton, insects, insect larvae and phytoplankton. It also eats a variety of other foods such as worms and small crustaceans (Spence et al. 2008). Many accept industrial food in the form of flakes when in aquariums. Studies with *D. rerio* have led to developments in fields as diverse as biology, oncology, toxicology, reproductive studies, teratology, genetics, neurobiology and environmental science, due to the numerous advantages that enable this fish as a model organism including completely sequenced genome; changes in behaviour are easily observed and tested; and similarities to mammals and humans in toxicity testing (Hill et al. 2005; Dahm 2006).

The use of industrial by-products such as vinasse for the production of fungal biomass is of great interest from the standpoint of sustainable production of the alcohol industry, as evidenced by Ferreira et al. (2010, 2011). Thus, this study aimed to verify the nutritional efficiency and toxicity of mycelia of the fungi *Pleurotus sajor-caju, P. albidus, P. flabellatus* and *P. ostreatus* cultivated in vinasse as a food source for *Danio rerio* and select the most promising species as a potential supplement to commercial feed.

## 2. Materials and methods

### 2.1. Vinasse

The vinasse was kindly donated by the Group Raizen-Costa Pinto Unit (a joint Venture formed between COSAN and SHELL companies), located in Piracicaba, SP, during the 2010–2011 crop season. The collection was made directly from the output of the fermentation vats, considered free of contaminants. Eighty litres of vinasse was collected in 20-L containers, presenting pH values between 4.7 and 4.8.

### 2.2. Fungi

Four edible basidiomycete fungi belonging to stock cultures of the Applied Ecology Laboratory of CENA/USP, Piracicaba, SP, were investigated, namely *Pleutotus sajor-caju* CCB 020, *P. albidus* CCB 068, *P. ostreatus* (shimeji) and *P. flabellatus* CCB 396. Fungi were inoculated into 250-mL Erlenmeyer flasks containing 100 mL of *in natura* vinasse without chemical changes or pretreatments. Six flasks per strain of fungus (four strains) were used plus six flasks as controls, all are properly autoclaved at 121°C for 15 minutes. After reaching room temperature, three plugs (1.0 cm Ø) containing fungal mycelia were inoculated into the culture medium. The flasks were incubated for 15 days in an incubator with an orbital rotation of 180 rpm, at 28°C ± 2°C in the dark.

### 2.3. Analysis of the mycelia of each fungus

To determine the bromatologic composition of the mycelia cultured for 15 days in vinasse, the fungi were lyophilised and macerated to a powder. The chemical analyses were performed following the official methods of the ‘Association of Official Analytical Chemists’ (1995).

The analyses were for dry matter (DM), mineral material or ashes (MM), organic matter (OM), crude protein (CP) as determined by nitrogen content released in the form of ammonia (micro-Kjeldahl method), neutral detergent fibre (NDF), acid detergent fibre (ADF), ethereal extract (EE) and Lignin.

### 2.4. Feed test with *Danio rerio*

The fungi grown for 15 days in vinasse were filtered with Whatman filter paper No.1 in kitassato with the aid of a vacuum pump and were then freeze-dried for 48 hours to produce the feed.

The culture medium used for cultivation of the fish was prepared according to ABNT NBR-15088 (2006), composed of Solution 1 (1.5 g of CaSO_4_.2H_2_O per litre of distilled water) and Solution 2 (0.20 g KCl, 4.8 g of NaHCO_3_ and 6.1 g MgSO_4_.7H_2_O per litre of distilled water). To a 20-L container, 400 mL of solution 1 and 200 mL of solution 2 were added; the container was then filled with distilled H_2_O. The pH was adjusted to 7.2–7.6 and the medium was kept under constant aeration for 24 hours to achieve oxygen saturation.

The fish were purchased from a commercial store and kept in an aquarium with a capacity of 25 L, with a substrate composed of fine sand and plants of the genus *Elodea* sp. to aid in aeration and shelter. They were kept in the aquaria for 2 weeks for acclimatisation and fed daily with commercial feed (TetraMin® brand Tropical flakes) under constant aeration and temperature of 22°C.

After this period, each fish was individually weighed on an analytical balance and its length measured on graph paper. They were then placed in a 250-mL beaker containing 225 mL culture medium with continuous oxygenation. Every 5 days, the culture medium was replaced to prevent accumulation of faecal matter on the bottom of the containers.

The diet offered daily to the fishes was prepared with the four species of *Pleurotus* in three concentrations: 100% lyophilised fungi, 50% lyophilised fungi +50% commercial diet and 100% commercial diet (control), with a photoperiod of 12-h light/dark and temperature of 22°C. For each concentration, five replicates were performed. Nine treatments were distributed in 50 beakers. The test lasted 28 days with individual measurement of body mass and size on the first and 28th days.

### 2.5. Statistical analysis

The results were analysed in Microsoft Office Excel 2007 and submitted for comparison of means by the Tukey test (5%), with the help of the System for Analysis and Separation of Means of Agricultural Experiment (SASM-Agri).

## 3. Results

### 3.1. Fungal biomass on vinasse

The fungal biomass yields are summarised in Table 1. Significant differences were found between the strains. The dry biomass varied from 8.20 g L^−1^ to 16.27 g L^−1^. The highest yield was obtained by *Pleurotus albidus*, reached 300 g L^−1^ of fresh or 16 g L^−1^ of dry biomass.

**Table 1.** Fresh and dry biomass (g L^−1^) of four strain of *Pleurotus* cultivated on vinasse for 15 days (28°C).

Strains	Fresh biomass	Dry biomass
*P. sajor-caju*	209.67^c^	12.73^c^
*P. albidus*	300.40^a^	16.27^a^
*P. flabellatus*	174.93^d^	8.20^d^
*P. ostreatus*	242.13^b^	13.27^b^

Note: Different letters indicate statistical difference by the Tukey test at a significance value of 0.05%.

### 3.2. Bromatological evaluation of *Pleurotus* sp. grown on vinasse

Mycelia from cultivation of each fungi in vinasse were freeze-dried and submitted to bromatological analysis (Table 2), thus obtaining their percentages of DM, based on the values of fresh weight (g kg^−1^) of each fungus.

**Table 2.** Bromatologic composition of the fungi cultivated on vinasse during 15 days. Dry matter (DM), mineral material (MM), organic matter (OM), crude protein (CP), ethereal extract (EE), neutral and acid detergent fibre (NDF and ADF) and lignin.

Fungi	DM	MM	OM	CP	EE	NDF	ADF	Lignin
*P. sajor-caju*	5.67	26.74	73.26	20.96	4.29	33.60	20.17	6.10
*P. albidus*	5.03	23.67	76.33	15.22	2.72	29.42	16.33	1.79
*P. flabellatus*	4.49	25.81	74.19	16.92	4.00	36.63	29.29	13.73
*P. ostreatus*	5.23	23.94	76.06	18.98	5.56	27.39	20.07	9.92
TetraMin T. Flakes*	–	20.50	79.50	47.00	10.0	3.00		–

Note: Parameter values expressed as % of dry matter. *TetraMin feed values were obtained from the packaging.

Chemical analysis showed the largest quantity of DM for the fungus *P. sajor-caju* (5.67%), followed by *P. ostreatus* (5.23%), *P. albidus* (5.03%) and *P. flabellatus* (4.49%) (Table 2). This parameter consists of MM and OM, which when added together, total 100% of the DM. Thus, fungi that show higher values of MM have lower amounts of OM in their composition and *vice versa. P. sajor-caju* showed a higher quantity of MM (26.74%) followed by *P. flabellatus* (25.81%). The value in the commercial feed was similar to those found in these fungi. In contrast, *P. albidus* and *P. ostreatus* fungi had high percentages of OM (76.33% and 76.06%, respectively). We also observed that the *P. albidus* showed the lowest percentage of lignin fibres in relation to other fungi (Table 2).

### 3.3. Feeding test using *D. rerio*

During testing, we observed that the fungi had good palatability to the fishes, that is, all given rations initially floated and then were promptly consumed. Food particles deposited on the bottom of the flask were consumed over time. No significant differences in the fungi preference were observed in the diets. However, with the exception of fungi *P. flabellatus*, the fishes gave preference to the commercial feed.

Fish showed weight gain in treatments with *P. albidus* (50%) and control (commercial feed). Treatment with *P. sajor caju* (50%) showed no difference in weight gain after 28 days and the other treatments showed weight loss, including treatments that used 50% of commercial feed (Table 3). A loss of fresh biomass of the fishes was observed with the diet *P. albidus* 100% (36.59%) and *P. sajor-caju* 100% (22.73%); in contrast, the fresh biomass increased by 7.89% in fishes fed with 50% *P. albidus* + 50% commercial diet. Similarly, the *P. sajor-caju* diet did not lead to a loss of weight, and when the results are compared with feed with 100% commercial diet, the gain of biomass was only 1.03% in the same period. For the other treatments, losses of biomass were around 15%.

**Table 3.** Biomass (g fresh^−1^ weight) and weight gain of *D. rerio* fed with *Pleurotus* sp. during 28 days. Negatives values mean weight loss. Means indicate a significant difference between the beginning and 28 days of feeding at the level of *P* < 0.05.

Fungi	Initial (g)	Final (g)	Weight gain (%)
*P. sajor-caju* (100%)	0.15^a^	0.11^a^	−22.73
*P. sajor-caju* (50%)	0.10^a^	0.10^a^	0.00
*P. albidus* (100%)	0.14^a^	0.09^b^	−36.59
*P. albidus* (50%)	0.13^a^	0.14^a^	7.89
*P. flabellatus* (100%)	0.21^a^	0.18^a^	−14.29
*P. flabellatus* (50%)	0.25^a^	0.21^a^	−13.51
*P. ostreatus* (100%)	0.13^a^	0.11^a^	−15.38
*P. ostreatus* (50%)	0.17^a^	0.15^a^	−10.00
Control	0.32^a^	0.33^a^	1.03

Note: Different letters indicate statistical difference by the Tukey test at a significance value of 0.05%.

**Figure 1.**
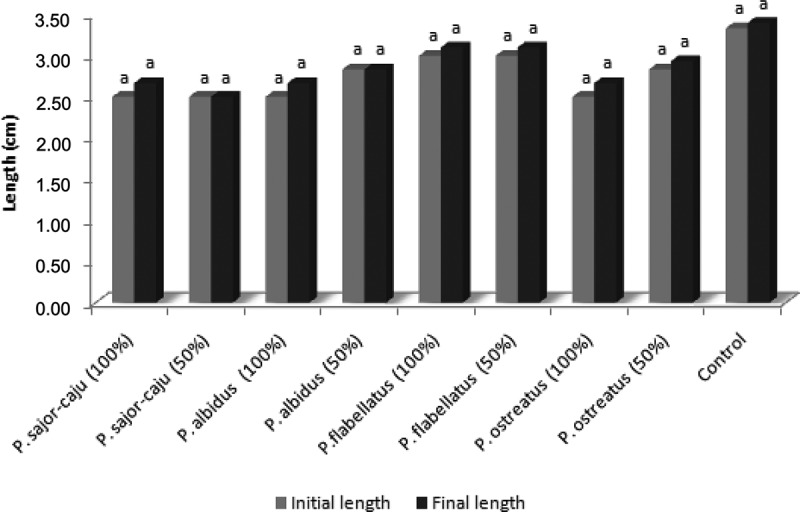
Length (cm) in the beginning and after 28 days of treatment with different diet. *P. sajor caju* 100% and 50% (fungi: commercial ration), *P. albidus* 100% and 50% (fungi: commercial ration), *P. flabellatus* 100% and 50% (fungi: commercial ration), *P. ostreatus* 100% and 50% (fungi: commercial ration) and control 100% commercial ration. Similar letters indicate the absence of statistical difference value of *P* < 0.05.

In addition to the biomass analyses, the length of each fish was performed. Statistically, there were no differences between the treatments (Figure 1). However, it can be seen that treatment with *Pleurotus* resulted in increased length of the fishes. Diet with 100% *P. sajor-caju, P. albidus* and *P. ostreatus* showed an increase of 6.8%, and treatments with *P. flabellatus* (50% and 100%) and *P. ostreatus* (50%) increased fish length by 3.3%, while an increase in size of only 2% was observed in the control fishes.

## 4. Discussion

In this study, the mycelia of fungi *Pleurotus sajor-caju, P. albidus, P. ostreatus* and *P. flabellatus* were inoculated in vinasse *in natura* for 15 days. The mycelia were lyophilised and then were offered as single food or as a dietary supplement for commercial fish. After 28 days, the nutritional efficiency and toxicity of mycelia were analysed.

### 4.1. Bromatological evaluation of *Pleurotus* sp. grown on vinasse

Confortin (2006) showed that there are similarities between the composition of mycelia produced in liquid medium and the fruiting body, thus allowing the results to be compared. We observed that the DM content found for the strains in this study (averaging 5.23%) was very close to those reported by Wadt (2008) when cultivating these fungi in pure vinasse, with values ranging from 4.42% for *P. albidus* to 8.85% for *P. sajor-caju*. In contrast, Sturion and Ranzani (2000) found higher values of DM for fruiting bodies of *Pleurotus* spp. (hiratake and shimeji), with an average of 10.27%.

The values for MM found in this study are higher than those found in the literature, with maximum values of 26.74%. Silva et al. (2007) obtained a maximum value of 6.4% for MM with *P. sajor-caju* grown on a substrate with high nitrogen content. Cultivating *P. sajor-caju* and *P. ostreatus* in banana straw, Bonatti et al. (2003) measured values of 5.58%, while Manzi et al. (1999) obtained values between 6.9% and 10.5% for *P. ostreatus* on banana leaf. The closest values found in the literature are those of Wadt (2008) in pure vinasse, with a maximum of 22.99% for *P. ostreatus* (shimeji). The same author reported that the high level of ash (MM) was a consequence of the use of vinasse as culture medium, confirming the high concentration of minerals present in this by-product.

The largest amounts of CP were found for *P. sajor-caju* (20.96%) and *P. ostreatus* (18.98%), while *P. flabellatus* and *P. albidus* did not reach values above 17%. These values are below the amount of CP present in the commercial concentrate (47%). Crude protein is an important parameter for the purposes of this study, since protein-rich foods are responsible for the largest fraction of the cost of feed in commercial fish-farming, as well as being a critical factor in additives to constituent feeds for aquaculture (Boscolo et al. 2001). The CP, as analysed here, reached a maximum value of 20.96% in the fungus *P. sajor-caju*, while the control (TetraMin T. Flakes®) had values around 47%. In the work of Wadt (2008), it was shown that when grown in vinasse, the highest value of CP was found for *P. albidus* CCB 068 (31.08%). Studies using other substrates for basidiomycetes have shown that values of CP vary depending on the culture medium in which the fungus is incubated. Silva et al. (2007) achieved 28% CP with *P. sajor-caju* in medium supplemented with high levels of nitrogen. Using the same fungus, Bonatti et al. (2003) obtained a maximum value of 16.86% CP using banana straw. On the same substrate, Ranzani and Sturion (1998) showed that the levels of protein in *P. sajor-caju* and *Pleurotus* sp. ‘Florida’ presented maximum values of 24.1%. The highest values for crude protein found in the literature were reported by Ragunathan and Swaminathan (2003) using the fungi *P. sajor-caju, P. platypus* and *P. citrinopileatus* grown in lignocellulolytic waste, obtaining maximum values of 25.63% to 44.30%.

The EE values are relatively low, ranging from 5.56% for *P. ostreatus* to 2.72% for *P. albidus*. The *P. sajor-caju* and *P. flabellatus* fungi showed mean values of 4% EE, while the commercial feed had values of 10%. The values obtained for the amount of EE were higher than those found by Wadt (2008) for fungi grown in vinasse, with values ranging from 0.36% to 4.69%, but are in agreement with the value of 6.50% for *P. ostreatus* (shimeji) presented by Furlani and Godoy (2007). For the fungus *P. sajor-caju*, low EE levels of 1.86% (in substrate with high nitrogen) were found by Sturion and Oetterer (1995). Again, with other nutrient sources used as feed, values were well above those found in the present paper. Fernandes et al. (2001) found 17% lipids in rice bran and 7% in soybean meal.

Regarding the fibre, the NDF fraction includes cellulose, hemicellulose and lignin as major components, while the fraction of ADF includes cellulose and lignin as primary components, in addition to varying quantities of ash and nitrogen compounds (Bianchini et al. 2011). It should be noted that NDF is more abundant than ADF, and the highest values for both parameters were found in the fungus *P. flabellatus*: 36.63% (NDF) and 29.29% (ADF). The lignin values obtained were also higher for *P. flabellatus* (13.73%), while the values for *P. sajor-caju, P. albidus* and *P. ostreatus* did not exceed 10% lignin. As already mentioned, lignin can be analysed with NDF and ADF, which explains the high values for *P. flabellatus*. The amount of lignin found in the mycelia probably occurs due to the presence of this compound in the vinasse resulting from processing of sugar cane.

Flores et al. (2008) examined fermentation by *Pleurotus* spp. in agro-industrial residues and obtained relatively high ADF values after fermentation of a mixture of straw, sawdust, bran and corn cob (65% ADF), or a mixture of straw and corn bran (70%) with the fungus *P. ostreatus*. Wadt (2008) presented similar values to those found in this work, with ADF values between 9.22% for *P. albidus* CCB 068 and 23.19 for *P. flabellatus* CCB 396. As for NDF, the same author obtained values ranging between 30.88% for *P. ostreatus* (shimeji) and 40.54% for *P. sajor-caju*.

Studies have also measured the values of total fibre contained in various basidiomycetes incubated in culture media. Bonatti et al. (2003) presented a value of 9.45% fibre in *P. sajor-caju*, while Furlani and Godoy (2007) achieved higher values with *P. ostreatus* (shimeji) in banana straw. Using lignocellulolytic waste as a substrate for *Pleurotus* sp., Ragunathan and Swaminathan (2003) achieved a value of 20.48% fibre. Compared to other kinds of food employed, such as soybean meal (6.45%) and canola meal (8.80%) (Gadioli et al. 2002), fungi have abundant fibre, whereas the commercial diet used as a control only contained 3%.

### 4.2. Feeding test using *D. rerio*

In this study, we observed an increase in weight gain of fish fed with commercial diet supplemented with *P. albidus*. The gain in weight of these fish was greater than the weight gain of fish fed only commercial feed. Siccard III et al. (2009) tested seven different types of feed on fish species of *Danio rerio*, including five commercial diets (400 AquaMax Grower ®, Cyclop-eeze ®, Flake Food Nutrafin Max ®, Tertra Pond Koi Vibrance ® and TetraMin Tropical Flakes ®) plus two other diets formulated by the author (diets A and B), adopted from Kovalenko et al. (2002) where diet A was formulated to contain 45.4% protein and 33.2% lipid, while diet B contained 41.4% protein and 33.2% lipid, in order to determine the effectiveness of their nutritional value. The fishes fed the special diets presented the highest growth (2.5 ± 1 cm) compared to the commercial diets (2.0 ± 2 cm). With regard to their biomass values, the highest value was for diet A, followed by Tropical Flakes TetraMin ® and diet B.

Other feeding sources were tested by Gadioli et al. (2002) who used canola meal to replace soybean meal in feed for alevins *Prochilodus lineatus* (Curimbatá), with the main parameter of analysis being the protein content in the meals, and obtained values for weight gain of 180–220%. Fernandes et al. (2001) also tested several mixtures of meals as protein sources for feeding juvenile pacu (*Piaractus mesopotamicus*), and despite the fact that there were no statistical differences among the protein sources tested, the fish that received fish meal as their main protein source showed a numerically smaller weight gain (38.97 g) than those who received soybean meal (43.75 g), while mixing fish meal plus soybean meal possibly gave the best results (51.75 g).

da Conceição et al. (2009) presented a review of new methodologies developed for studies of nutrition in fish larvae, following the premise that survival rates are often low or variable, and quality issues are often experienced, so that nutrition and growth potential are not maximised, and concluded that the biological basis for the development and nutrition of fish larvae has improved greatly, especially with the use of isotopic techniques that use markers and functional genomics, though understanding of the physiology of nutrition and nutritional value is still limited.

Nutrition is now known to be an important determinant of disease progression in all rodent models, and authors are now required to report the specific diet used in these investigations. However, this variable and its consequences have not been included in research efforts with fish such as *D. rerio* (Siccard III et al. 2009). In the absence of an understanding and standardisation of food consumption, it is necessary to seek new research sources of nutrition and to understand their effects on the development and health of fish, especially those intended for human consumption.

Even though biomass loss occurred in this study, although this was not significant, the values of survival of the fish were 100% in all treatments, indicating that the fungi tested are non-toxic and can be included in the diet of animals as nutritional supplements. The loss of biomass during the test may have been due to stress factors, such as physical space, light and in particular handling during weighing and changing the culture medium of fishes. Although growth rates are not necessarily indicative of health of the fish, Siccard III et al. (2009) suggest that the *D. rerio* reacts to the quantity and/or quality of specific nutrients required for normal growth rates. Diets offered may influence other physiological processes, including those associated with endocrine, neurological, reproductive or immunologic systems (Goede & Barton 1990).

In the absence of understanding and standardisation of food consumption, researches are necessary in the seeking of new sources of nutrition and its effects on the development and health of fishes, especially animals destined for human consumption.

## 5. Conclusions

From the results obtained in this study, we conclude that the four species of fungi tested, *Pleurotus sajor-caju, Pleurotus albidus, Pleurotus ostreatus* and *Pleurotus flabellatus*, had satisfactory mycelial growth in vinasse. The bromatological analysis of mycelia showed similar values with the commercial rations being *P. albidus* the most promising species tested, and the feeding test with *Danio rerio* showed no toxic effects, indicating that de dry mycelia can be used as possible dietary supplements for fishes.
